# What Do the Parents of Children With Disabilities Look for in a Dedicated Orthodontic Clinic: A Grounded‐Theory Study

**DOI:** 10.1111/scd.70114

**Published:** 2025-11-17

**Authors:** Julia Huber, Valbona Soumas, Gregory S. Antonarakis

**Affiliations:** ^1^ Division of Orthodontics University Clinics of Dental Medicine University of Geneva Geneva Switzerland

**Keywords:** children with disabilities, grounded theory, orthodontic treatment, parental satisfaction

## Abstract

**Aims:**

The present qualitative study aims to explore the needs of families with children with disabilities, regarding their orthodontic care in a dedicated university orthodontic clinic.

**Methods:**

Thirteen parents of children with disabilities, followed in a University orthodontic clinic dedicated to individuals with disabilities, were selected to participate in the present study. A grounded‐theory approach was used to conduct informal in‐depth interviews, which were digitally recorded and transcribed verbatim. Interview transcriptions were analyzed to develop a theoretical proposition grounded in real‐world observations.

**Results:**

A core category was identified, namely the aspiration of parents of children with disabilites to afford a positive experience for their child. In association with the core category, six other categories emerged: location, accessibility, and a welcoming environment; convenience and availability; an empathetic team; good communication; appropriate and modern equipment; and quality of treatment outcome.

**Conclusions:**

The families of children with disabilities seek personalized and high‐quality orthodontic care in a favorable environment with a welcoming, empathetic, and communicative team. Gaining a clearer understanding of what families value in clinics and teams that provide orthodontic care for children with disabilties can help inform meaningful and positive improvements to these dedicated services.

## 1 Introduction

Orthodontic treatment needs in the population of children with disabilities are high, and more than half of these patients have severe malocclusion [1, 2]. Despite this high treatment need, however, many parents of children with disabilities struggle to find qualified health professionals who are willing to provide care for their children [3]. A recent study in Switzerland, based on questionnaires sent to specialist orthodontists, found that 84% of orthodontists see children with disabilities. However, most see very few of these patients, who appear to receive treatment less often than the general population [4]. In addition to that, their need for orthodontic care is often more complex and takes longer. Orthodontists usually limit the number of these patients in their practice because they feel they lack the specific skills, appropriate training, time in a busy orthodontic practice, and the oral health status is often considered not conducive for allowing orthodontic treatment to be recommended [4].

In many health systems, most of the provision of orthodontic services is done privately, which implies that parents often have to search for a private practitioner willing and able to see their child independently. This mission is all the more laborious for parents of children with disabilities, given that they may require different management with regard to factors such as empathy, time, and accessibility [5]. Those with disabilities face complex physical, behavioral, and multidimensional barriers in accessing appropriate services, and these issues reduce access to care and even more for families outside urban settings.

When an orthodontist decides to offer treatment to a child with a disability, several obstacles may occur throughout the treatment. The most frequent obstacle is maintaining regular appointments, and given that these families often struggle to find an orthodontist able to treat their child, they often need to travel greater distances for appointments to see those who are willing and able to treat this population. Secondly, dental hygiene may be problematic, as patients and families are frequently somewhat ignorant about appropriate oral hygiene protocols, which may be further complicated by poor manual dexterity [6].

Improving the care provided to families of children with disabilities requires their direct participation and input as one of the key stakeholders. This calls for research with this in mind, allowing the orthodontic community to adapt to the needs of those concerned. To date, little has been done to address the research need in this direction. A recent study from Chile looking into the barriers and facilitators of orthodontic treatment in teenagers with developmental disabilities [7] states that this lack of research could explain the lack of knowledge about improving education, experience, and the patients’ quality of life in relation to orthodontic treatment. A certain fear of treatment is observed and shared by both the patients and the caretakers [7].

Patients with disabilities and their parents seek orthodontic treatment primarily for reasons such as improving facial appearance, but other motivating factors include dental health, mastication, and speech [8]. Parents expect their children to be treated to improve oral health and function while realizing that achieving a perfect result may not be realistic. Treating these children is often more complex, both with regard to the initial malocclusion but also related to challenges with collaboration, communication, and oral hygiene, therefore finishing with an improved but not perfect orthodontic outcome can be considered sufficient. Aiming to improve the child's self‐esteem should be seen as one of the important benefits of providing such a treatment [8].

In order to gain further insight into the views of parents of children with disabilities concerning their orthodontic management, qualitative methods offer several advantages in being able to discover the participants’ points of view in unbiased ways. While quantitative methods help gain objective and generalizable knowledge, individual perspectives as well as other relevant and critical subjective issues may not emerge sufficiently [9]. In oral health research, the use of qualitative research such as grounded‐theory approaches puts the participants’ viewpoint at the center, and this helps in achieving a more thorough understanding of the subject in question [10].

Previous research has highlighted the need to not only improve the access of children with disability to dental care, but also the benefits of setting up specialized clinics which would help in doing so while improving prophylaxis and care [3].

The present study aims to use qualitative methods to increase awareness of the needs of the parents of children with disabilities in relation to their orthodontic uptake in a specialized orthodontic clinic.

## 2 Materials and Methods

This study was conducted in accordance with the ethical principles outlined in the Declaration of Helsinki. As the study did not involve health‐related or identifiable data, and data analyzed were extracted from anonymized content, according to local regulations formal ethical approval was not required. The reporting of the present study was carried out in accordance with the standards for reporting qualitative research (SPQR) [11].

### 2.1 Qualitative Approach and Researcher Characteristics

Grounded theory, a sociological theory often used to assess healthcare quality in qualitative studies [12], was chosen as the methodological basis of the present study. Data collection was conducted through interviews in this context. Developed by Glaser and Strauss in 1967 for qualitative research, grounded theory utilizes an inductive method and aligns with the interpretivism paradigm [13]. Unlike deductive approaches, which test hypotheses through data gathering, grounded theory aims to create theoretical insights from the data [14]. This methodological approach not only guided how the data were collected but also influenced the dynamics between the researcher and participants during discussions, ensuring both objectivity and depth in the analysis process.

The researcher who conducted the interviews had no prior relationship with the participants apart from a single phone call to arrange the appointment.

### 2.2 Study Group

The study sample was intended to be parents of patients followed in the specialized university clinic for individuals with disabilities (within the University Clinics of Dental Medicine, University of Geneva, Switzerland). Eligible candidates were parents who met the following criteria: they had a child with a disability, their child was receiving orthodontic treatment at the aforementioned clinic, and they had attended at least two orthodontic appointments for their child. A representative sample was strived for, encompassing parents of children with different conditions, and of different ages and both males and females, as well as parents of both sexes (mothers and fathers). The only exclusion criterion was if the parents did not speak either English or French, or if the person accompanying the child was not a parent but another legal guardian. The recruitment of more parents as part of the sampling process was stopped once data saturation was achieved, as is customary when carrying out such interview‐based grounded‐theory studies.

All interviews were to be treated anonymously without recording any identifying information (name, date of birth, postal code) of the children or the parents in question, and all interviews were conducted confidentially and with the interviewee's consent.

### 2.3 Data Collection Methods

Between December 2022 and April 2023, data were collected through open‐ended interviews in which the interviewees guided the conversation. The content of the interviews was analyzed gradually as the interviews were carried out, in order to establish the data saturation point. Data saturation was defined as the point at which no new information emerged from the additional interviews.

### 2.4 Interview Procedure

Contact with the various parents was initiated via informal telephone calls, and appointments were scheduled based on the availability of those who provided consent. The interviews, lasting between 10 and 30 min, were conducted in person in a room at the university without the presence of any people other than the interviewer and interviewee, or via videoconference or telephone. Open discussions were anonymous and recorded using a voice recorder with the parents' consent.

Prior to the series of interviews, a pilot discussion was held with a parent who met the study's inclusion criteria, but whose child was not receiving orthodontic treatment at the university clinic. This parent, an activist involved in supporting Swiss individuals with disabilities, generously shared the challenges of the parents in managing their child's medical care. In the same way, they offered perspectives on the ideal structure of a medical appointment and suggestions for sensitively addressing the topic of disability. This instructive conversation helped inform the interviewer on how to carry out the interview while being sufficiently empathetic and understanding.

The interviews were open‐ended, all starting with the broad question: “What are your experiences and expectations regarding your visit to our dedicated orthodontic clinic?” Each participant was then given the opportunity to discuss freely and raise any topics or themes they considered important. The interviewer, when necessary, asked follow‐up questions for probing and clarification, always based on what the participant had already shared, in order to better understand their experiences and expectations. This approach ensured consistency across the interviews while leaving space for participants to express their perspectives in their own words.

### 2.5 Units of Study

Thirteen parents of children with disabilities, namely six mothers and seven fathers, were interviewed until data saturation was reached. Among them, five were parents of girls and eight of boys, aged between 1 and 26 years (Table 1). In total, 18 parents had been contacted, 5 of them having chosen not to participate in the interview process.

**TABLE 1:** Patient demographics, whose parents participated in the study.

Patient #	Age (years)	Sex	Relative	Handicap
1	13	F	Father	Down syndrome
2	18	M	Mother	Myopathy
3	12	M	Mother	Myopathy
4	17	F	Father	Down syndrome
5	17	M	Mother	Autism spectrum disorder
6	14	M	Mother	Down syndrome
7	12	M	Father	Autism spectrum disorder
8	13	F	Father	Behavioral disorder
9	10	M	Father	Down syndrome
10	16	M	Mother	Myopathy
11	12	F	Father	Visual impairment
12	26	M	Mother	Complex craniofacial disorder
13	1	F	Father	Complex craniofacial disorder

The interviewees were parents of individuals with seven distinct pathologies: four with Down syndrome, three with myopathies, two with autism spectrum disorder, one with a behavioral disorder (but without a specific diagnosis), one with visual impairment, and two with complex craniofacial anomalies.

### 2.6 Data Processing and Analysis

After each discussion, a verbatim transcript of the audio recordings was written and analyzed to identify key categories. The data coding process involves three steps: open coding, which is the initial step, focuses on identifying what participants describe as their actions, feelings, or experiences [15]; axial coding, which consists of developing connections between concepts that emerge across the different interviews; and selective coding, the final step, aimed at identifying the main category along with other significant categories.

To structure the analysis, data from each interview were organized into data tables specific to the interviewer, along with one experienced clinician who also carried out the data analysis independently. This approach facilitated the identification of commonalities across the interviews, allowing various themes to emerge. To ensure objectivity and clarity, the two researchers, along with the senior researcher, applied a triangulation approach by comparing the respective data, which led to the identification of distinct categories [16]. The analysis was conducted manually. Each researcher noted emerging themes independently, then compared observations collaboratively to identify recurring themes and ensure consistency, without using specialized qualitative data‐analysis software.

To minimize bias in this qualitative study, the two examiners independently listened to and analyzed the discussions as they proceeded, highlighting the relevant data without consulting each other [17].

## 3 Results

**FIGURE 1:**
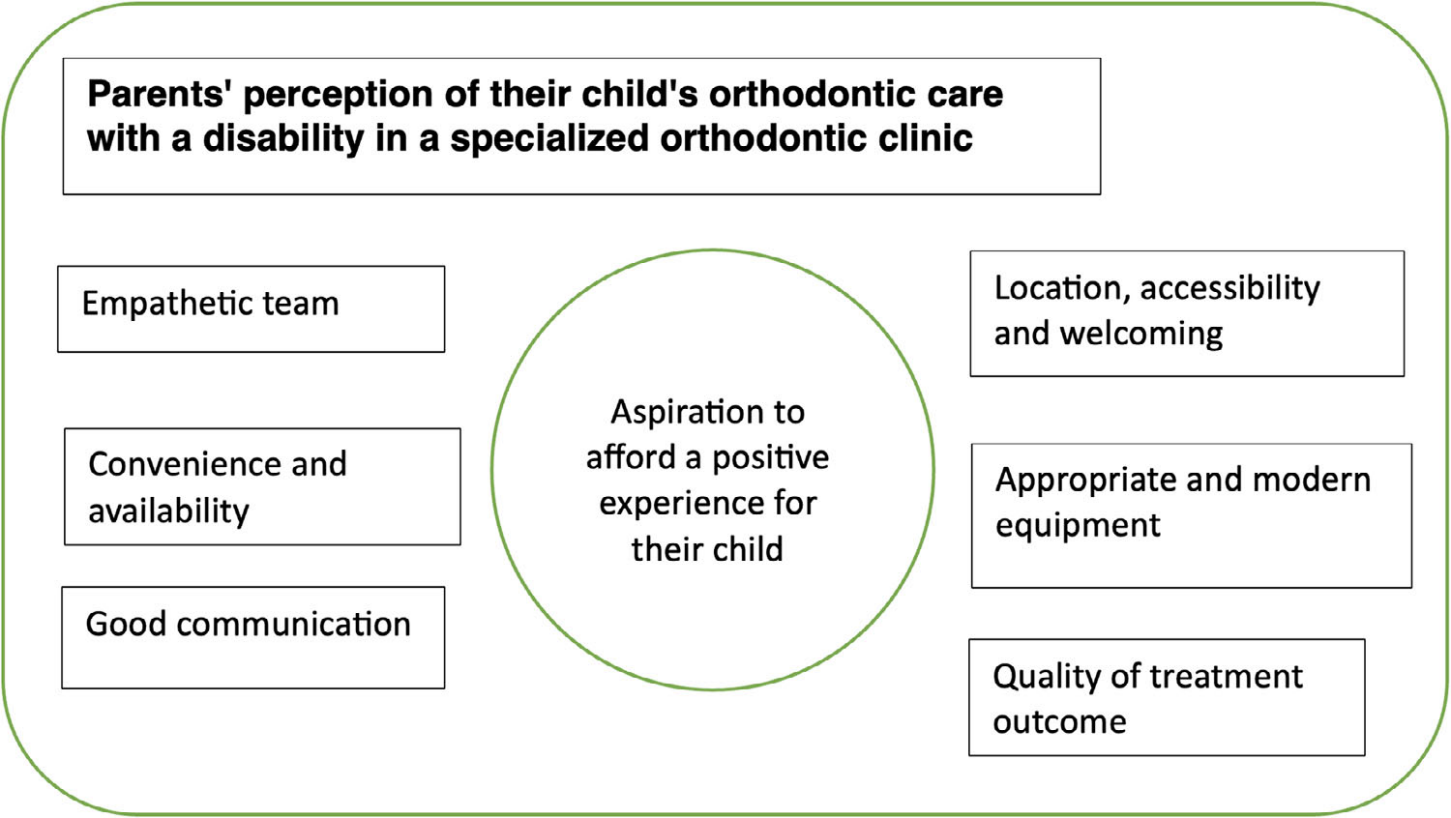
Grounded theory model illustrating the core category at the center and the six related categories reflecting the perceptions of parents of a child with a disability regarding their orthodontic care in a specialized orthodontic clinic.

Data from the conducted interviews resulted in the emergence of one core category and six other related categories (Figure 1). The core category describes the parents’ aspiration to afford a positive experience for their child regarding their orthodontic care. The six other categories identified were: location, accessibility and a welcoming team; appropriate and modern equipment; convenience and availability of appointments and staff; good communication; an empathetic team; and the quality of the treatment outcome.

### 3.1 Core Category: Aspiration to Afford a Positive Experience for Their Child

The most crucial point for the parents was their child's feelings about the follow‐up and care provided at the specialized orthodontic clinic. Parents emphasized the importance of their child seeing the same caregivers consistently during appointments. With the habit of regular follow‐ups, children became comfortable returning to the clinic, with some even expressing enjoyment. Many of them felt at ease in the clinic and appreciated the consistency and gentleness of the staff.

*The assistant is always the same, which is great for the child's confidence. For example, even if the doctor is running a bit late due to other commitments, the same assistant is there to reassure my child*.

Parents frequently noted that their child showed little to no fear or anxiety about the orthodontic visits after a certain point. Pain was also rarely experienced by the children receiving care. Parents appreciated the clinical staff's approach, which placed the child at the center of care, fostering increased therapeutic adherence.

Both the children and their caregivers received clear and sufficient explanations about the progress of the orthodontic treatment. This communication helped strengthen the therapeutic relationship.

### 3.2 Location, Accessibility, and a Welcoming Team

The interviews revealed that the welcome during the first and subsequent visits at the reception was crucial to the child's comfort, ease, and well‐being. Parents explained that addressing the child by their first name and speaking in a gentle tone helped them feel at ease. The atmosphere was described as friendly, and the positive mood significantly reduced the child's apprehension about being examined and receiving care.

*From the reception to the health professionals, we are always well received, everyone's in a good mood, and it doesn't feel like a stressful place at all. I also find that the schedules are pretty good. Sure, there are delays occasionally, but it's nothing like some dental practices where you end up waiting forever*.

*My child is happy. He's even become friends with one of the security staff, who greets him every time, which makes him very proud to be recognized. Things always go smoothly on site. There's also a playful side. He gets to try on gloves, and when he leaves, he feels good about the experience. There's no pain or trauma to deal with afterward, so for him, it's positive*.

The location of the clinic and its accessibility were also important for families. The clinic is centrally located within the city and with access to an underground parking area with ample parking spaces. Wheelchair access was also deemed an essential feature with an entrance suitable for individuals with mobility difficulties. Parents were comforted by the fact that security guards were also always on site, and they often helped the families if needed with simple logistic issues, such as helping a child get through a crowded reception area. Accessibility was also important for the families, with regard to wheelchair access to the clinic and to the dedicated dental unit, which was spacious and had room for the child regardless of their mobility situation and the presence of multiple family members.

### 3.3 Appropriate and Modern Equipment

All parents shared their opinion on the importance of appropriate and modern equipment. The clinic's modern design was a key factor in the appreciation of the parents. Many parents highlighted how clean and well‐lit the clinic was. The waiting room, described as large and spacious, was particularly appreciated.

A further key point raised by numerous parents during the interviews conducted concerned the equipment. Parents found the clinic to be very well‐equipped with state‐of‐the‐art technological devices, such as intraoral scanners and three‐dimensional photographic cameras. They expressed gratitude for having everything in one place, including various radiography machines.

*Everything is on‐site; for example, we just had to go a bit further within the same building for an x‐ray appointment*.

*It's very good because we find the separate clinical boxes very convenient. Since our child is immunosuppressed, we have to be extra careful about viruses and bacteria. Having insulated boxes is very reassuring for us. We feel like we're in a spotless place, and it suits us very well*.

### 3.4 Convenience and Availability of Appointments and Staff

All the interviewees highlighted the team's availability. The efficient management of the emergency department reassured parents, who appreciated being able to book spontaneous appointments when problems were encountered.

*Even in an emergency, they would see my child quickly, or another practitioner would step in if they couldn't. That's really important*.

Although parents often contact the dedicated specialized clinic through the general secretariat or via email, some mentioned that having a direct telephone line dedicated to the specialized clinic would be more convenient. Subsequent to this remark, a dedicated telephone was attributed to the specialized clinic.

In some cases, particularly involving insurance matters, parents felt relieved that the orthodontist and dental assistant handled the complex administrative procedures.

*What's really nice and helpful for us is the administrative side, especially the management of the insurance issues, which is handled so well. It spares us from having to deal with all these complex procedures ourselves. They took care of everything, explained exactly what to do, and prepared us in advance for any forms we needed to fill out. That's something we really appreciated, as it's usually so complicated*.

However, many parents noted that the clinic lacked visibility, as most had discovered it through word of mouth or recommendations from their doctors or other dentists.

Finally, the excellent timeliness and availability of the appointments were frequently praised. Many parents expressed satisfaction with the clinic's appointment management, noting short waiting times and appropriately timed sessions that were never too long. The parents also expressed gratitude for this, as their daily lives are often filled with numerous medical appointments, and finding appointments at the orthodontist that could suit their busy schedules was refreshing.

### 3.5 Good Communication

The relationship between families and the staff was another critical element in the orthodontic care of children with disabilities. For all the parents in this study, the interactions met their expectations. They expressed feeling at ease from the very first exchanges with the staff. To foster this bond of trust, parents emphasized the importance of receiving clear explanations about clinical procedures and the treatment plan before the sessions.

It was also noted that it is essential for the orthodontist to address the child first, treating them like any other child, without focusing on their disability as the primary concern.

Throughout the treatment, the parents found it valuable for the orthodontist to show and explain the progress of the treatment to the child, keeping them motivated. The taking of frequent orthodontic records was appreciated as this helped communication with the families and the children.

Involving the child in clinical procedures, allowing them to touch and test certain instruments and giving them responsibilities, particularly regarding their own follow‐up, was thought by the parents to strengthen the child's autonomy, intrinsic motivation, and adherence to the treatment.

According to the parents, certain moments during a clinical session can leave a lasting impression on the child. These memorable moments are primarily those when the child receives compliments or congratulations from the healthcare team.

*The team does their best to make my daughter feel comfortable working with them. For example, they sometimes play music or tell stories*.

*My daughter needs to be able to talk things through and get explanations. She also needs to laugh. It's important for her to understand what's happening in the moment. Sometimes, my daughter tries to tell stories, and as I mentioned, her language skills aren't very strong, but the team understands her perfectly, which really puts her at ease*.

### 3.6 Empathetic Team

Most parents shared their opinions on the clinic's overall atmosphere and, more specifically, on the team that comprises it. Some parents paid close attention to interprofessional and interpersonal relationships. These relationships were described as natural, straightforward, and respectful. The staff were perceived as relaxed, cheerful, and fostering mutual respect toward each other as well as toward the families, creating a climate of harmony and understanding. The perception of hierarchy within the team was not felt, and the parents appreciated this aspect. As a result, no parent reported a stressful or anxiety‐inducing atmosphere transmitted to their child or to themselves.

It was often noted that the health professionals take the time needed during consultations, without looking as if they were in a rush to move on to the next patient. They show genuine interest, ask patients about their lives, and occasionally share anecdotes about their own. According to the parents, a touch of humor in these interactions is often appreciated and helps the patient feel at ease.

*My son likes it, and he's not anxious at all. Before, he didn't really like going to the dentist, but now it's almost a pleasure. He's happy to see the doctor and gets along well with him. They talk a lot, especially about football, because my son loves it. He really enjoys these conversations*.

*They talk about sports. The doctor has found topics my son likes, and they discuss those, so he feels comfortable. I think it's important to get to know the patient. If you can connect with them and talk about the things they enjoy, the experience goes by much quicker than just focusing on the clinical task. It's also about being social; being attentive helps things go more smoothly than simply doing the job. That's what makes the difference between a good doctor and one who's just doing their job*.

### 3.7 Quality of Treatment Outcome

All the interviewees highlighted that the excellent quality of care is a fundamental pillar shaping parents' opinions of the specialized clinic.

According to the parents, the key criteria for evaluating the quality of care include the child's well‐being, the professionalism of the care team, empathy toward the patient and their companions, the academic environment, efficient time management, and the treatment outcomes. Some parents particularly appreciated the interprofessional collaborations among the various health specialists involved in their child's care. This spared them from having to repeatedly update the clinic on their child's medical history.

The university setting was perceived as reassuring, with parents feeling that care was serious, up‐to‐date and innovative. The lack of strict hierarchical relationships among staff members was described as healthy and constructive. Parents observed the interactions between professors, consultant clinicians, assistants, and students, noting that these exchanges were open and free from judgment.

Care was reported to be personalized and tailored to each patient's specific needs, including adjustments for unforeseen circumstances.

Finally, all the children who completed their orthodontic treatment were satisfied with the results, as were their parents.

*I expect the professionals to do their job well, and so far, that's exactly what's happening. Everything is going very well. At the moment, I have no complaints*.

*It's important not to feel like patients are just being brought in to make money, which we felt with other orthodontists in the private sector. Also, the work is well done and progresses each time. After the conversation with the assistant, asking questions and understanding what is happening is really important. Here, we have everything: a great team, assistants who explain things and are kind, and good quality treatment*.

## 4 Discussion

The present study focused on a clinic specializing in orthodontic care for children with disabilities, using grounded theory to explore parents’ perceptions and gather qualitative data. This approach allowed for in‐depth exploration [18] whereby open conversations were useful in collecting valuable insights, particularly regarding the operations of a recently established specialized orthodontic clinic [15].

With limited hindsight into this question, this study provides valuable perspectives on the essential needs of families, highlighting aspects of care that might not initially be apparent during treatment, yet are fundamental to the oral health‐related quality of life. From the thirteen open interviews conducted, seven distinct categories emerged, including one core category that consistently appeared across all testimonies. Participant testimonies serve as the fundamental unit of analysis in grounded‐theory studies [19].

The core category identified was the parents aspiration to afford a positive experience for their child. Throughout the discussions, all parents emphasized that their child's opinion and well‐being were the most crucial factors influencing their perception of the specialized orthodontic clinic. Children generally reported feeling comfortable and confident in the clinic. Several parents noted that when their child feels good, it positively impacts their own well‐being [20].

This research suggests that a child's comfort during orthodontic visits is closely tied to parental satisfaction with the care provided. Effective communication and a psychologically‐adapted approach from the practitioner and their team are key to ensuring this comfort. A systematic review about psychology and communication skills in orthodontic practice further supports these findings, highlighting their significant role in patient cooperation and the overall treatment experience [21].

The lives of these young patients are marked by numerous medical appointments, adding to an already challenging daily routine, and frequent consultations are not always pleasant for these children. Parents expressed that numerous medical appointments could lead to understandable irritability and loss of patience in the child. Studies have shown that access to oral health care remains particularly difficult for young patients with disabilities, often because their medical needs are not prioritized in the same way as those of other patients [22]. This highlights the importance of a thoughtful therapeutic approach, one that allows sufficient time for each consultation while paradoxically being both efficient and swift, fostering a strong and trusting relationship with patients. A warm and compassionate welcome at the specialized clinic was highlighted as essential to reassuring patients from the moment they enter the building and throughout the initial reception.

Parents emphasized the importance of the location of the clinic within a university, which acted as a significant source of reassurance. The university setting was perceived as a guarantee of safety, quality of care, and access to multidisciplinary expertise supported by state‐of‐the‐art equipment. Parents appreciated the convenience of having comprehensive dental care (including oral hygiene, conservative, prosthetic, and surgical treatments) along with state‐of‐the‐art radiography facilities all within the same clinic. Overall, they expressed satisfaction with appointment management, noting short waiting times and immediate attention in case of emergencies as very important.

As witnessed by the participants, fostering a strong relationship between the patient and the orthodontist is essential. The practitioner's empathy and patience encourage patient compliance, and children appreciate being treated as active participants in their care, with responsibilities assigned to them. Parents valued the practice of explaining each clinical step in advance and outlining what would follow. Visual aids, such as photographs, radiographs, and before‐and‐after dental impressions, were particularly helpful for enhancing understanding [23]. Additionally, it was noted that the orthodontist should engage directly with the child, treating them as they would treat all other children, using humor when possible, and showing interest in their hobbies and preferences [24].

Parents were aware that their involvement is crucial for long‐term treatment success, as they must take on new responsibilities throughout their child's orthodontic journey [25]. Equally important to parental motivation is the relationship between the patient and the entire care team, which plays a vital role in the child's well‐being, and parents appreciated seeing a healthy and constructive hierarchical relationship within the university. The alleviation of concerns regarding administrative procedures, particularly those related to disability insurance, was greatly appreciated by parents. These findings are consistent with those of a recent systematic review highlighting the challenges faced by children with disabilities in accessing appropriate dental care and underscore the importance of a supportive, well‐coordinated and multidisciplinary clinical environment [26].

While the present study offers valuable insights, its findings mainly apply to a specialized university orthodontic setting. The presence of multidisciplinary teams and advanced care facilities may not be found in all clinics or dental practices. However, the key principles such as the welcoming atmosphere and supportive environment, clear communication, the orthodontist's verbal and non‐verbal behavior, and child‐centered care, can be applied to improve specialized pediatric and orthodontic dental services in general [27]. It is important to note that the categories identified were inferred from parents’ accounts, and not assessed directly. Since the patients were minors or under guardianship, their experiences were shared through their parents. Despite this, the data remain highly relevant to dental professionals, offering valuable insights and practical tools for clinicians caring for children with special needs [7].

## 5 Conclusions

This study assessed the needs of parents of children with disabilities with regard to the provision of their orthodontic care, using a grounded‐theory approach. Seven key categories emerged from the participant interviews, providing valuable insight for specialized orthodontic care and underscoring the importance of adopting a holistic approach in orthodontic care, focusing not only on clinical outcomes but also on the emotional and psychological aspects that influence both patient and parental satisfaction.

## Funding

No funding was obtained for the present study.

## Ethics Statement

This study was conducted in accordance with the ethical principles outlined in the Declaration of Helsinki. As the study did not involve health‐related or identifiable data, and the data analyzed were extracted from anonymized content, formal ethical approval was not required according to local regulations.

## Conflicts of Interest

The authors declare no conflicts of interest.

## Data Availability

The data that support the findings of this study are available from the corresponding author upon reasonable request.
